# Violence against children: what can we do?

**DOI:** 10.1016/j.lansea.2025.100541

**Published:** 2025-02-10

**Authors:** The Lancet Regional Health – Southeast Asia

Violence against children can be physical, mental, or sexual; injury and abuse, neglect and negligent treatment, maltreatment, or exploitation. It can happen in any setting—at home, in the community, school, workplace, and online. And for many, the perpetrators are someone they trust—their parents, caregivers, family, neighbours, or teachers. However, children living with disabilities or with HIV/AIDS, those living in extreme poverty or in institutional care, and those who are homeless or are migrants, refugees, or asylum seekers are at higher risk.

A systematic review in 2020 estimated that over a quarter (27.8%) to nearly half (47.6%) of children experienced severe forms of physical violence by their caregivers in the south Asia region. In most countries of the south Asia region, 1 in 10 children reported experiencing sexual violence in childhood, which could be an underestimate due to harmful social norms, deep-rooted stigma, and fear of discrimination and marginalisation, perpetuated by law enforcement, judiciary, and the community at large. The latest UNICEF report estimates that southern Asia has one of the largest numbers of rape and sexual assault victims in the world. In the 2020 systematic review, emotional violence was reported by children even in the absence of other forms of violence. In the south Asia region, extreme poverty can contribute additional impacts of mental and physical violence caused by child marriages and child labour. In this part of the world, violent discipline is a socially accepted norm; a study of a nationally representative dataset in Bhutan found that the most common forms of physical violence against children were committed in the context of corporal punishment in schools. Recent data continue to show strong links between experiencing violence in childhood and negative mental health outcomes, and this may be a driver of the high adolescent self-harm rates in the region. In India, self-harm is the leading cause of death among adolescent girls and the second leading cause of death among adolescent boys.

Violence against children has lifelong impacts on the health and wellbeing of children, families, communities, and nations. Children who experience violence early in their lives are more likely to drop out of education, have difficulty finding jobs, and be victims or perpetrators of violence in later life, which perpetuates intergenerational violence. The south Asia region is also witness to continued sociopolitical conflicts, rapid economic changes, and economic and health emergencies such as COVID-19 and a recent surge in natural disasters attributed to climate change. These have resulted in an intergenerational impact on the physical and mental wellbeing of children and adolescents. Despite several pieces of legislation and legal provisions formulated to control this detestable crime, violence against children remains widespread. Target 16.2 of the 2030 Agenda for Sustainable Development is to “end abuse, exploitation, trafficking and all forms of violence against, and torture of, children”.

Violence against children can be prevented. Preventing and responding to violence against children requires systematic efforts that address risk and protective factors at individual, relationship, community, and society levels. WHO developed a technical package called *INSPIRE: Seven strategies for ending violence against children* to guide ministries and governments. Several countries have committed to include at least four strategies in their national action plans. The 2023–2030 Strategy to End Violence against Children and the accompanying 2023–2024 Action Plan also share strategies and actions to free the world of crime and violence against children. National governments are working to address the response services for victims, including child protection services, clinical services for sexual violence victims, victim identification and referral, and victim mental health services. All actors—governments, international and local organisations, civil society, private and public sector institutions, schools, parents, children, and young people—play an important role in preventing and responding to crime and violence against children. More than a decade after the implementation of the Protection of Children from Sexual Offences (POCSO) Act in India, a landmark piece of legislation to protect children from sexual abuse, there has been no decline in crimes against children in the country, and there are still more acquittals than convictions. There is a need to strengthen preventive interventions, including a change in norms and values; safe environments; parental and caregiver support; income and economic strengthening; and education and life skills, in addition to strengthening capacity building of judicial and law enforcement agencies. In this issue of *The Lancet Regional Health – Southeast Asia*, we publish an intervention that utilises entertainment-education to promote positive parenting and reduce violence against children. This study was conducted in a province of Thailand that shares a border with Myanmar and has seen armed conflict for decades.

Now more than ever we need strategies to increase capacity building, augmenting skills and reforming social norms and structures related to preventing violence against children. We need to stop normalising the exploitation and assault of children at the hands of more powerful people. We need to include children and adolescents in policy making and programmes, everything that impacts their present and future. There is a dire need to reform our stance to handle not only children's safety and security but also how to best use our resources to make childhood the best experience of their lives. Children, their caregivers, and wider communities need to be more aware and educated about the rights of children, available support, and how to use these support services. Researchers now identify the need to listen to children's and youth voices and to strengthen their agency, for both research and the development of interventions, prevention programmes, and policies. Governments need to reform judicial systems and law enforcement machinery to be child-friendly, responsive, supportive, and reflective of their commitment to timely justice and effective redressal. They should also cater to the growing needs of children, keeping up with the ever-evolving face of crime. Children should not be looked upon merely as our future, people who will fulfil our aspirations, but as individuals in a very important stage of their lives—childhood, which is supposed to be beautiful, full of fairies and unicorns and not the horrific sounds of bullets and assault haunting their dreams and later lives.

